# Spinal Radiographic Progression in Patients with Ankylosing Spondylitis Treated with TNF-α Blocking Therapy: A Prospective Longitudinal Observational Cohort Study

**DOI:** 10.1371/journal.pone.0122693

**Published:** 2015-04-16

**Authors:** Fiona Maas, Anneke Spoorenberg, Elisabeth Brouwer, Reinhard Bos, Monique Efde, Rizwana N. Chaudhry, Nic J. G. M. Veeger, Peter M. A. van Ooijen, Rinze Wolf, Hendrika Bootsma, Eveline van der Veer, Suzanne Arends

**Affiliations:** 1 Department of Rheumatology and Clinical Immunology, University of Groningen, University Medical Center Groningen, Groningen, The Netherlands; 2 Department of Rheumatology, Medical Center Leeuwarden, Leeuwarden, The Netherlands; 3 Department of Epidemiology, University of Groningen, University Medical Center Groningen, Groningen, The Netherlands; 4 Department of Radiology, University of Groningen, University Medical Center Groningen, Groningen, The Netherlands; 5 Department of Radiology, Medical Center Leeuwarden, Leeuwarden, The Netherlands; 6 Department of Laboratory Medicine, University of Groningen, University Medical Center Groningen, Groningen, The Netherlands; Oregon Health & Science University, UNITED STATES

## Abstract

**Objectives:**

To evaluate spinal radiographic damage over time and to explore the associations of radiographic progression with patient characteristics and clinical assessments including disease activity in ankylosing spondylitis (AS) patients treated with tumor necrosis factor-alpha (TNF-α) blocking therapy in daily clinical practice.

**Methods:**

Consecutive outpatients from the Groningen Leeuwarden AS (GLAS) cohort were included based on the availability of cervical and lumbar radiographs before start of TNF-α blocking therapy and after 2, 4, and/or 6 years of follow-up. Clinical data were assessed at the same time points. Radiographs were scored by two independent readers using the modified Stoke AS Spine Score (mSASSS). Spinal radiographic progression in relation to clinical assessments was analyzed using generalized estimating equations.

**Results:**

176 AS patients were included, 58% had syndesmophytes at baseline. Median mSASSS increased significantly from 10.7 (IQR: 4.6–24.0) at baseline to 14.8 (IQR: 7.9–32.8) at 6 years. At the group level, spinal radiographic progression was linear with a mean progression rate of 1.3 mSASSS units per 2 years. Both spinal radiographic damage at baseline and radiographic progression were highly variable between AS patients. Male gender, older age, longer disease duration, higher BMI, longer smoking duration, high CRP, and high ASDAS were significantly associated with syndesmophytes at baseline. Significantly more radiographic progression was seen in patients with versus without syndesmophytes (2.0 vs. 0.5 mSASSS units per 2 years) and in patients >40 versus ≤40 years of age (1.8 vs. 0.7 mSASSS units per 2 years). No longitudinal associations between radiographic progression and clinical assessments were found.

**Conclusions:**

This prospective longitudinal observational cohort study in daily clinical practice shows overall slow and linear spinal radiographic progression in AS patients treated with TNF-α blocking therapy. At the individual level, progression was highly variable. Patients with syndesmophytes at baseline showed a 4-fold higher radiographic progression rate than patients without syndesmophytes.

## Introduction

Ankylosing spondylitis (AS) is a chronic rheumatic inflammatory disorder which usually begins before the fourth decade of life. AS is characterized by inflammation in combination with new bone formation and bone loss. The disease mainly affects the axial skeleton and causes pain, stiffness, and impaired functioning of the spine. The disease course is found to be highly variable between AS patients. Excessive bone formation is an important disease outcome of AS. In the spine, this comprises the formation of syndesmophytes which may lead to complete fusion of the spine, resulting in a so-called ‘bamboo spine’. In most AS patients, it takes years from the first disease symptoms to manifestations of bone formation on radiographs [1]. Therefore, long-term follow-up is needed to investigate radiographic progression.

Tumor necrosis factor-alpha (TNF-α) blocking therapy leads to a clear improvement in disease activity, functional outcome measures, and quality of life in the majority of AS patients who do not respond to conventional treatment [1]. However, variable results have been reported regarding the effect of TNF-α blocking therapy on the development of spinal radiographic damage in AS. Multiple open-label extension studies did not show a significant difference in spinal radiographic progression after 2 years of TNF-α blocking therapy compared to TNF-α blocker naive AS patients from historical cohorts [2–5]. Two other open-label extension studies could not demonstrate an inhibition of spinal radiographic progression during 4 years of TNF-α blocking therapy [6,7]. However, in a retrospective study in only 22 AS patients, diminished radiographic progression was found after 4 to 8 years of TNF-α blocking therapy compared to AS patients from a historical cohort [8]. Furthermore, a large prospective longitudinal observational study with 1.5 to 9 years of follow-up reported that TNF-α blocker exposure (2.5 ± 2.8 years) was associated with less spinal radiographic progression [9].

These findings triggered the debate about the effect of TNF-α blocking therapy and the relationship between disease activity and spinal radiographic progression in AS. In previous cross-sectional and longitudinal studies in AS patients with a large variability in disease duration, disease activity, and treatment regimens, disease activity at baseline and over time were associated with spinal radiographic damage and progression [10–12]. Also, elevated inflammatory markers at baseline were found to be associated with the presence of syndesmophytes at baseline and with radiographic progression in AS patients and in early axial spondyloarthritis (SpA) [10,11]. Very recently, a longitudinal association between the AS Disease Activity Score (ASDAS) and radiographic progression was observed during 12 years of follow-up in a large cohort of AS patients mainly treated with non-steroidal anti-inflammatory drugs (NSAIDs) [12].

The aim of this prospective longitudinal cohort study was to evaluate spinal radiographic damage over time and to explore the associations of radiographic progression with patient characteristics and clinical assessments including disease activity in AS patients treated with TNF-α blocking therapy in daily clinical practice.

## Methods

The present analysis was based on data from the Groningen Leeuwarden Ankylosing Spondylitis (GLAS) cohort. GLAS is an ongoing prospective longitudinal observational cohort study in the northern part of the Netherlands. Since November 2004, this cohort included consecutive AS outpatients who started TNF-α blocking therapy at the University Medical Center Groningen (UMCG) or the Medical Center Leeuwarden (MCL) because of active disease [13]. All patients were over 18 years of age, fulfilled the modified New York criteria for AS [14], and the ASAS criteria to start TNF-α blocking therapy (active disease defined as Bath AS Disease Activity Index (BASDAI) ≥4 and/or based on expert opinion) [15].

The choice of the TNF-α blocking agent (infliximab, etanercept, or adalimumab) was based on the judgment of the treating rheumatologist and/or the specific preference of the patient. As described previously, the standard regimen for infliximab was 5 mg/kg intravenously at 0, 2, 6 weeks and then every 8 weeks, for etanercept 50 mg (once) or 25 mg (twice) subcutaneous injection every week, and for adalimumab 40 mg subcutaneous injection every two weeks [16].

Patients were clinically evaluated at baseline, after 3 months, and then every 6 months according to a fixed protocol. Disease activity was measured at each follow-up visit and treatment continuation was based on BASDAI improvement (≥50% or two units compared with baseline) and/or expert opinion. Patients were allowed to switch between different TNF-α blocking agents and to receive concomitant medication as usual in daily clinical practice. Type, dose, and frequency of TNF-α blocking therapy were recorded at all follow-up visits. Temporary stop was registered and the total duration of exposure to TNF-α blocking therapy was expressed as the percentage of follow-up time.

Patients included in the analysis started with TNF-α blocking therapy between 2004 and October 2011 and had lateral radiographs of the cervical and lumbar spine available at baseline and after at least 1 follow-up visit at 2, 4 and/or 6 years.

The GLAS cohort was approved by the local ethics committees of the MCL and the UMCG. All patients provided written informed consent according to the Declaration of Helsinki.

### Data collection

Baseline characteristics included: gender, age, symptom duration, time since diagnosis, HLA-B27 status, history of smoking (duration in years), and history of extra-articular manifestations.

At baseline and at each follow-up visit, disease activity was assessed with BASDAI [18], ASDAS_CRP_ [19,20], physician’s and patient’s global assessment (GDA), C-reactive protein (CRP), and erythrocyte sedimentation rate (ESR). Since all patients had high disease activity at baseline and in order to analyze whether baseline disease activity status was associated with spinal radiographic progression, cut-off values for very high disease activity as defined in previous studies were used to stratify patients: BASDAI >6 [12], ASDAS >3.5 [21], physician’s and patient’s GDA >6 [21], CRP >10 mg/L, and ESR >20 mm/hr [11]. Furthermore, body weight and height were assessed to calculate body mass index (BMI), NSAID use was recorded, and ASAS-NSAID index was calculated [17].

### Assessments of spinal radiographic damage

Lateral radiographs of the cervical and lumbar spine were independently scored by two trained readers (FM and RC). In order to blind readers for patient characteristics and time sequence, all identifying information including exam dates were removed from the radiographs. Radiographs were scored using the modified Stoke AS Spine Score (mSASSS). The anterior corners of lower C2 until upper Th1 and lower Th12 until upper S1 were scored for the presence of erosions, sclerosis, and/or squaring (1 point per vertebral site), non-bridging syndesmophytes (2 points per site), and bridging syndesmophytes (complete bridging of vertebrae; 3 points per site). The mSASSS was calculated as the sum of the scores of all individual sites (range 0–72). Patients with complete spinal ankylosis (mSASSS of 72) at baseline were excluded since no radiographic progression could occur in these patients. If ≤3 scores of vertebral sites were missing, the scores of these sites were substituted by the mean score of the vertebrae of the corresponding spinal segment, as proposed by Wanders *et al*. [22,23]. If >3 scores of vertebral sites were missing, the radiograph was excluded from the analysis. Radiographs were reassessed if the mSASSS total score of both readers differed by >5 units. When the discrepancy of >5 units persisted after reassessment, consensus was reached. The average mSASSS total score of both readers was used for the analysis.

Inter-observer reliability between readers for baseline mSASSS was very good, with an intraclass correlation coefficient (ICC; two-way mixed effects model, single measures, absolute agreement; before reassessment) of 0.987 (95% confidence interval (CI): 0.982–0.991). Inter-observer reliability for mSASSS change scores was moderate to good with ICC’s of 0.690 (95% CI: 0.596–0.765) for 0–2 year interval, 0.690 (95% CI: 0.545–0.794) for 2–4 year interval, and 0.400 (95% CI: 0.110–0.626) for 4–6 years interval. Bland-Altman plots revealed no systematic error. The mean difference in progression scores between the two readers was 0.1 (95% CI -3.2−3.4) for all 2-years intervals (S1 Fig).

Presence of syndesmophytes at baseline was defined when both readers scored a non-bridging or bridging syndesmophyte (≥2 points) at one or more vertebral sites. Inter-observer reliability for presence of syndesmophytes was very good with Cohen’s kappa of 0.89 (95% CI 0.83−0.96) and absolute agreement of 95%.

Definitions of spinal radiographic progression according to Baraliakos *et al*. were used to distinguish between slow progression (<2 mSASSS units within 2 years), moderate progression (2 to 5 mSASSS units within 2 years), and fast progression (>5 mSASSS units within 2 years) [24].

### Statistical analysis

Results were expressed as mean ± SD or median (interquartile range (IQR)) for normally distributed and non-normally distributed data, respectively. Independent samples T-test, Mann-Whitney U test, Chi-Square test, and Fisher Exact test were used to compare differences in baseline characteristics between groups.

Generalized estimating equations (GEE) was used to analyze spinal radiographic progression over time within subjects and to calculate mean radiographic progression rate at the group level. Because correlations of spinal radiographic damage were approximately equal at different time points, the exchangeable correlation structure was used. Different models of time (linear, quadratic, cubic, square, logarithmic, and exponential) were used to investigate whether time was linear or non-linear associated with radiographic progression. In case residuals were non-normally distributed, parameters were transformed (log or square root) before entered into the equation.

In the baseline analysis, interactions between time and the following patient characteristics and baseline clinical assessments were tested: gender, age, symptom duration, time since diagnosis, HLA-B27 status, BMI, duration of smoking, NSAID use, disease activity (BASDAI, ASDAS, physician’s and patient’s GDA, CRP, ESR), and presence of syndesmophytes. If interaction effects with time were found (p-values ≤0.05), the mean radiographic progression rate was calculated after stratification into subgroups based on clinically relevant or median values.

In the longitudinal analysis, the relationship between radiographic progression and disease activity, BMI, and NSAID use over time was investigated with an autoregressive marginal time-lag model. This model investigates the influence of disease activity at the start of a 2-year interval (eg. BASDAI_t_), BMI at the start of a 2-year interval (BMI_t_), or mean cumulative NSAID use during a 2-year period (ASAS-NSAID_t−t+1_) on the radiographic score at the end of a 2-year interval (mSASSS_t+1_), adjusted for the radiographic score at the start of this interval (mSASSS_t_) so radiographic progression was modeled. The following models were tested: mSASSS_t+1_ modeled by mSASSS_t_ and BASDAI_t_, ASDAS_t_, physician’s GDA_t_, patient’s GDA_t_, CRP_t_, ESR_t_. BMI_t_, and ASAS-NSAID_t−t+1_,

Statistical analysis was performed with IBM SPSS Statistics 22 (SPSS, Chicago, IL, USA). P values ≤0.05 were considered statistically significant.

## Results

**Fig 1:**
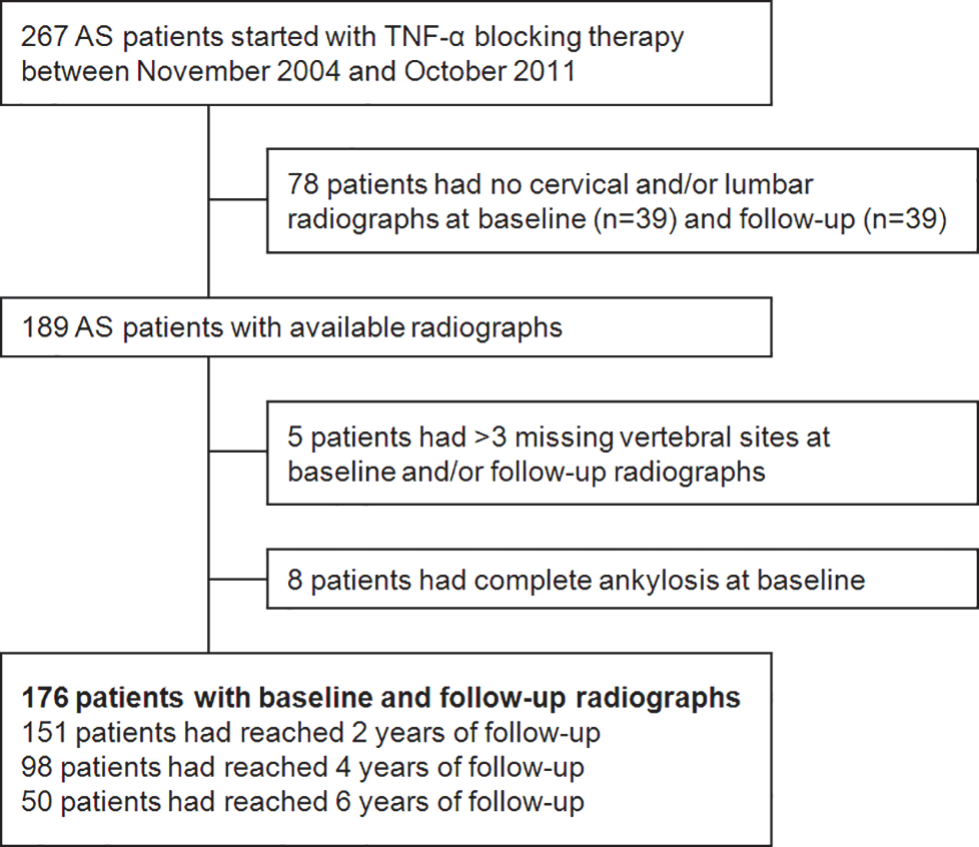
Flowchart of AS patients included in the analysis.

In total, 176 of the 267 AS patients who started with TNF-α blocking therapy between November 2004 and October 2011 were included in the analysis (Fig 1). Baseline characteristics of included patients were comparable to those who were excluded because of missing radiographs (n = 78) or >3 missing vertebral edges (n = 5) at baseline or at follow-up, except for symptom duration (median 14 vs. 17 years, p<0.05). Eight patients were excluded because of complete spinal ankylosis at baseline. These patients were older (mean 55 vs. 42 years, p<0.01) and had longer symptom duration (median 38 vs. 14 years, p<0.01).

Of the 176 included patients, 69% were male, mean age was 42 ± 11 years, median symptom duration 14 years (IQR: 7–24), and 77% were HLA-B27 positive (Table 1). History of inflammatory bowel disease, uveitis, psoriasis, and peripheral arthritis were seen in 11%, 32%, 7%, and 34% of the patients, respectively.

**Table 1:** Baseline characteristics of the total AS study population and stratified by the presence or absence of syndesmophytes at baseline.

		Baseline syndemophytes		
	All patients (n = 176)	Present (n = 102)	Absent (n = 74)	p-value
**Male gender**	121 (69)	78 (77)	43 (58)	**0.009**
**Age (yrs)**	42.3 ± 11.1	46.8 ± 10.1	36.2 ± 9.4	**<0.001**
**Symptom duration (yrs)**	14 (7–23)	18 (10–25)	10 (5–17)	**<0.001**
**Time since diagnosis (yrs)**	5 (1–14)	8 (1–19)	3 (1–11)	**0.011**
**HLA-B27+**	134 (77)	77 (76)	57 (77)	0.903
**BMI (kg/m** ^**2**^)	26.4 ± 4.1	27.2 ± 4.1	25.2 ± 3.7	**0.004**
**Smoking (yrs)**	12 (0–22)	16 (0–25)	7 (0–16)	**0.020**
**NSAID use**	130 (74)	79 (78)	51 (69)	0.203
**ASAS-NSAID index (0–100)**	50 (0–100)	50 (0–100)	40 (0–100)	0.602
**BASDAI (0–10)**	6.1 ± 1.6	6.1 ± 1.5	6.0 ± 1.7	0.929
BASDAI >6	84 (48)	52 (51)	32 (43)	0.310
**ASDAS**_**CRP**_	3.7 ± 0.8	3.8 ± 0.7	3.6 ± 0.8	0.150
ASDAS >3.5	104 (60)	68 (68)	36 (49)	**0.013**
**Physician’s GDA (0–10)**	4 (3–6)	4 (3–6)	4 (3–6)	0.701
Physician’s GDA >6	34 (20)	23 (23)	11 (16)	0.238
**Patient’s GDA (0–10)**	7 (5–8)	7 (5–8)	7 (6–8)	0.280
Patient’s GDA >6	105 (60)	56 (55)	49 (66)	0.151
**CRP (mg/L)**	12 (4–22)	14 (6–22)	9 (4–21)	0.141
CRP >10 mg/L	97 (56)	63 (62)	34 (47)	**0.038**
**ESR (mm/hr)**	21 (10–34)	20 (10–34)	21 (9–34)	0.832
ESR >20 mm/hr	87 (50)	49 (49)	38 (52)	0.691
**mSASSS (range 0–72)**	11 (5–24)	21 (12–38)	4 (2–7)	**<0.001**

Values are presented as number of patients (%), mean ± SD, or median (IQR).AS: ankylosing spondylitis; HLA: human leukocyte antigen; BMI: body mass index; NSAID: non-steroidal anti-inflammatory drug; ASAS: Assessment of SpondyloArthritis international Society; BASDAI: Bath AS Disease Activity Index; ASDAS: AS Disease Activity Score; GDA: global disease activity; CRP: C-reactive protein; ESR: erythrocyte sedimentation rate; mSASSS: modified Stoke AS Spine Score.

All patients had high disease activity at baseline (91% BASDAI ≥4, 99% ASDAS ≥2.1, and 68% CRP ≥6mg/L). Twenty-seven (15%) patients started with infliximab, 110 (63%) with etanercept, and 39 (22%) with adalimumab. During follow-up, 45 (26%) switched to another TNF-α blocking agent. Patients were exposed to TNF-α blocking therapy for 97% of the follow-up time (IQR: 83%- 100%).

### Spinal radiographic damage and clinical assessments before the start of TNF-α blocking therapy

At baseline, median mSASSS was 11 (IQR: 5–24) and 102 (58%) patients had at least one syndesmophyte according to both readers. Patients with syndesmophytes at baseline were more frequently male, older, had longer symptom and diagnosis duration, higher BMI, longer duration of smoking, and had more often very high disease activity based on ASDAS (>3.5) and CRP (>10 mg/L) (Table 1).

### Spinal radiographic progression during TNF-α blocking therapy and baseline clinical assessments

Mean clinical follow-up time was 3.8 ± 1.8 years (range 1–7). During this period, 176, 151, 98, and 50 patients had mSASSS data available at baseline and after 2, 4, and 6 years of follow-up, respectively. Baseline characteristics were comparable in all these groups, only a significantly longer symptom duration and higher ASAS-NSAID index were seen in patients with 6 years data (Table 1 and S1 Table).

Median mSASSS increased significantly from 10.7 (IQR: 4.6–24.0) at baseline to 14.8 (IQR: 7.9–32.8) at 6 years (Table 2). At the group level, a linear time model revealed the best fit for the data. Mean progression rate was estimated at 1.3 mSASSS units per 2 years.

**Table 2:** mSASSS status and progression scores of AS patients who started with TNF-α blocking therapy.

Status scores	Baseline (n = 176)	2 year (n = 151)	4 year (n = 98)	6 year (n = 50)
Mean mSASSS	16.9 ± 16.7	17.5 ± 17.2	21.4 ± 18.5	21.4 ± 18.7
Median mSASSS	10.7 (4.6–24.0)	10.9 (4.6–25.5)	15.0 (5.6–32.1)	14.8 (7.9–32.8)
**Progression scores**		**0–2 year (n = 151)**	**2–4 year (n = 75)**	**4–6 year (n = 42)**
Mean change mSASSS		1.3 ± 3.2	1.4 ± 2.8	1.3 ± 2.0
Median change mSASSS		0.8 (-0.5–2.1)	1.2 (-0.6–2.8)	1.0 (-0.1–2.8)
<2 mSASSS units/2 years (slow)		106 (70)	44 (59)	27 (64)
2–5 mSASSS units/2 years (moderate)		27 (18)	25 (33)	13 (31)
>5 mSASSS units/2 years (fast)		18 (12)	6 (8)	2 (5)

Values are presented as number of patients (%), mean ± SD or median (IQR).

mSASSS: modified stoke AS spine score.

**Fig 2:**
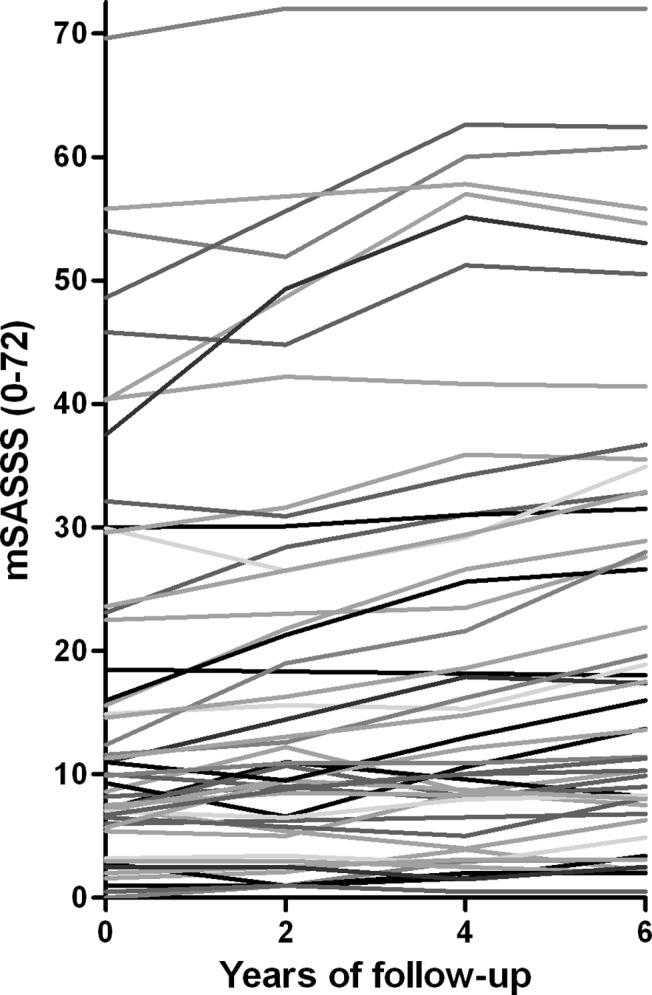
Spinal radiographic progression at patient level of AS patients with 6 years of follow-up (n = 50).

At the individual level, both spinal radiographic damage at baseline and radiographic progression over time were highly variable between AS patients (Fig 2). During the 2-years intervals, no or slow progression was found in 59–70%, moderate progression in 18–33%, and fast progression in 5–12% of the patients (Table 2).

The presence of syndesmophytes and older age were significantly associated with spinal radiographic progression. Patients with syndesmophytes at baseline had a 4-fold higher radiographic progression rate than patients without syndesmophytes (2.0 vs. 0.5 mSASSS units per 2 years). This increased progression rate also applies for patients with only 1 syndesmophyte compared to patients without syndesmophytes (1.8 vs. 0.5 mSASSS units per 2 years). Patients >40 years of age showed a 2.5-fold higher radiographic progression rate than patients ≤40 years (1.8 vs. 0.7 mSASSS units per 2 years) (Table 3).

**Table 3:** Effect of time and time interactions with baseline characteristics on spinal radiographic progression.

	β (95% CI)	p-value	Intervals	n
**Time**	1.25 (1.10–1.40)	**<0.001**	475	176
Time*gender		0.104	475	176
Time*age		**0.045**	475	176
Age ≤40 years	0.68 (0.49–0.90)		206	75
Age >40 years	1.78 (1.64–1.93)		269	101
Time*symptom duration		0.565	448	166
Time*time since diagnosis		0.516	465	171
Time*HLA-B27		0.855	473	175
Time*BMI		0.221	360	141
Time*smoking		0.117	362	133
Time*NSAID use		0.246	475	176
Time*ASAS-NSAID index		0.668	381	130
Time*BASDAI		0.526	475	176
Time*ASDAS		0.764	467	173
Time*Physician’s GDA		0.123	466	172
Time*Patient’s GDA		0.506	473	175
Time*CRP		0.375	469	174
Time*ESR		0.339	466	173
Time*syndesmophytes		**0.022**	475	176
Without syndesmophytes	0.52 (0.39–0.66)		203	74
With syndesmophytes	2.02 (1.88–2.17)		272	102

See Table 1 for abbreviations.

### Spinal radiographic progression and clinical assessments during TNF-α blocking therapy

Disease activity improved significantly during TNF-α blocking therapy. From baseline to 3 months, mean BASDAI improved from 6.1 to 3.2, mean ASDAS from 3.7 to 2.1, median physician’s GDA from 4 to 2, median patient’s GDA from 7 to 3, median CRP from 12 to 3 mg/L, and median ESR from 21 to 6 mm/hr (all p<0.001). These improvements remained stable during long-term follow-up (data not shown). Mean BMI showed a small increase during follow-up, from 26.4 at baseline to 26.6 at 6 years (p<0.05). NSAID use decreased significantly from 74% at baseline to 41%, 37%, and 25% at 2, 4, and 6 years, respectively (p<0.001). Mean cumulative NSAID intake according to the ASAS-NSAID index decreased from 24.3 during the first 2 years to 14.4 and 9.2 during the 2–4 and 4–6 years time intervals, respectively (p<0.001).

During TNF-α blocking therapy, no significant longitudinal associations were found between spinal radiographic progression and disease activity, BMI, or NSAID use over time (Table 4). Also the change in disease activity during the first 3 to 6 months, remission at 6 months (e.g. ASDAS<1.3), prolonged remission (ASDAS<1.3 for at least 2 consecutive visits), and change in NSAID use during the first 2 years were not significantly associated with spinal radiographic progression (data not shown). Only a trend was observed for the longitudinal association of CRP and ESR levels with radiographic progression (Table 4).

**Table 4:** Longitudinal analysis of the relationship between spinal radiographic progression and disease activity, BMI, and NSAID use.

	β (95% CI)	p-value	Intervals	n
**Previous mSASSS**	1.02 (1.00–1.04)	**<0.001**	268	159
**BASDAI**	-0.02 (-0.15–0.10)	0.737	259	159
**ASDAS**	0.24 (-0.08–0.55)	0.143	255	158
**Physician’s GDA**	-0.05 (-0.19–0.09)	0.497	264	156
**Patient’s GDA**	-0.03 (-0.16–0.10)	0.637	267	159
**CRP**	0.03 (0.00–0.06)	0.071	255	158
**ESR**	0.03 (0.00–0.06)	0.056	252	158
**BMI**	-0.02 (-0.09–0.05)	0.596	266	181
**ASAS-NSAID index**	0.00 (-0.01–0.01)	0.905	196	87

See Table 1 for abbreviations.

## Discussion

This observational longitudinal cohort study prospectively investigated spinal radiographic damage over time and the associations of radiographic progression with patient characteristics and clinical assessments including disease activity in 176 AS patients treated with TNF-α blocking therapy in daily clinical practice. Spinal radiographic progression was linear at the group level with a mean progression rate of 1.3 mSASSS units per 2 years. This indicates that, on average, AS patients treated with TNF-α blocking therapy showed slow radiographic progression according to the definitions of Baraliakos *et al*. (<2 mSASSS units per 2 years) [24]. Radiographic progression was also linear during 12 years of follow-up in a large cohort of AS patients mainly treated with NSAIDs. In this cohort, the mean progression rate was estimated at 2.0 mSASSS units per 2 years [12,25]. Although the mean 2-year progression rate found in our patients treated with TNF-α blocking agents was lower, no conclusions can be made since a direct comparison between the two cohorts is lacking. Other studies in AS patients treated with TNF-α blocking therapy for 2–8 years showed variable mSASSS progression scores with a minimum of 0.4 and a maximum of 1.8 mSASSS units per 2 years [2–8].

Before start of TNF-α blocking therapy, all AS patients had high disease activity and more than half (58%) had syndesmophytes, which is comparable to the results of previous studies. In these previous studies, the proportion of patients with syndesmophytes at baseline varied from 30% in ‘early’ AS (≤10 years symptom duration) [11], 47–58% in AS patients with a variable disease activity status [25] to 55–61% in AS patients with active disease before start of TNF-α blocking therapy [7,8]. In accordance with earlier findings, we found that male gender, older age, elevated CRP levels, but also longer symptom and diagnosis duration, longer smoking duration, and ASDAS >3.5 were significantly associated with the presence of syndesmophytes at baseline [10,26]. Additionally, we found that patients with high disease activity and syndesmophytes at baseline had significantly higher BMI which suggests an association between disease activity, BMI, and radiographic damage. However, this could not be confirmed in the longitudinal analyses.

At the individual level, spinal radiographic progression was highly variable. The mean mSASSS progression rate was 4-fold higher in patients with syndesmophytes at baseline and 2.5-fold higher in patients >40 years of age. These findings indicate that radiographic progression during the treatment of TNF-α blocking therapy mainly occurs in older AS patients and in patients with more advanced disease. Previous studies with different treatment regimens also identified the presence of syndesmophytes at baseline as the most important predictor for the development of more radiographic damage in both ‘early’ axial SpA [11] and longstanding AS [2,10,24,27].

In our analyses, none of the disease activity assessments at baseline and over time were significantly associated with spinal radiographic progression. This is probably due to the low variability in disease activity since all patients had high disease activity before start of TNF-α blocking therapy and stable low disease activity during treatment. Moreover, the mean change in mSASSS at the group level was small, which makes it difficult to observe significant differences. This was also confirmed by the low observed regression coefficients of the time-lagged autoregressive GEE models. Historical longitudinal observational cohort studies in AS patients that have found significant relationships between disease activity and radiographic progression included patients with a high variability in disease activity status and treatment regimens [11,12]. In the Outcome in AS International Study (OASIS), patients with very high disease activity (ASDAS >3.5) over time showed an additional increase of 2.3 mSASSS unts per 2 years compared to patients with inactive disease (ASDAS <1.3) [12]. In another analysis of the same cohort, ESR was significantly associated with the development of new syndesmophytes after 4 years of follow-up (OR 1.03, 95% CI: 1.00–1.07, p<0.05) [10]. In 210 early axial SpA patients from the German Spondyloarthritis Inception Cohort (GESPIC), elevated ESR levels at baseline (>20 mm/hr) and time-averaged elevated CRP levels over 2 years (>6 mg/L) were significantly associated with spinal radiographic progression during 2 years of follow-up [11].

Previous studies in AS reported a positive effect of continuous use of NSAIDs on the reduction of radiographic progression [28,29]. In our study, NSAID use decreased rapidly over time resulting in very low ASAS-NSAID index scores, as expected in patients starting TNF-α blocking agents. Only 10% of the patients had a cumulative NSAID intake of ≥50 according to the ASAS-NSAID index and no effect on radiographic progression could be found. Furthermore, follow-up data on smoking was not available in this study and therefore we could not include smoking in the longitudinal model to investigate this influence on spinal radiographic progression.

In the present study the reading of the radiographs was done without known time sequence which may lead to negative and smaller progression rates than when the reading was done in chronological time order [30]. Furthermore, it was not possible to draw conclusions about the effect of TNF-α blocking therapy on spinal radiographic progression, since AS patients without TNF-α blocking therapy were not included.

## Conclusion

This large prospective observational cohort study in AS patients treated with TNF-α blocking therapy in daily clinical practice showed that spinal radiographic progression was overall slow and linear at the group level. At the individual level, radiographic progression was highly variable. Patients with syndesmophytes at baseline had a 4-fold increased radiographic progression rate and patients >40 years of age had a 2.5-fold increased radiographic progression rate. No longitudinal associations between radiographic progression and clinical assessments were found. A direct longitudinal comparison between cohorts with long-term follow-up and large study populations of AS patients treated with and without long-term TNF-α blocking therapy is required to evaluate the effect of this treatment and to further investigate the relationship between clinical assessment (e.g. disease activity) and spinal radiographic progression.

## Supporting Information

S1 Fig Bland-Altman plot representing the reliability of the mSASSS change scores. (TIF)

S1 Table Baseline characteristics of AS patients with available 2, 4, or 6 years mSASSS data. (PDF)
