# Supplementary figures and images for: Spinal Radiographic Progression in Patients with Ankylosing Spondylitis Treated with TNF-α Blocking Therapy: A Prospective Longitudinal Observational Cohort Study
Source: PLoS One. 2015 Apr 16;10(4):e0122693. doi: 10.1371/journal.pone.0122693 (PMC4400173; doi:10.1371/journal.pone.0122693)

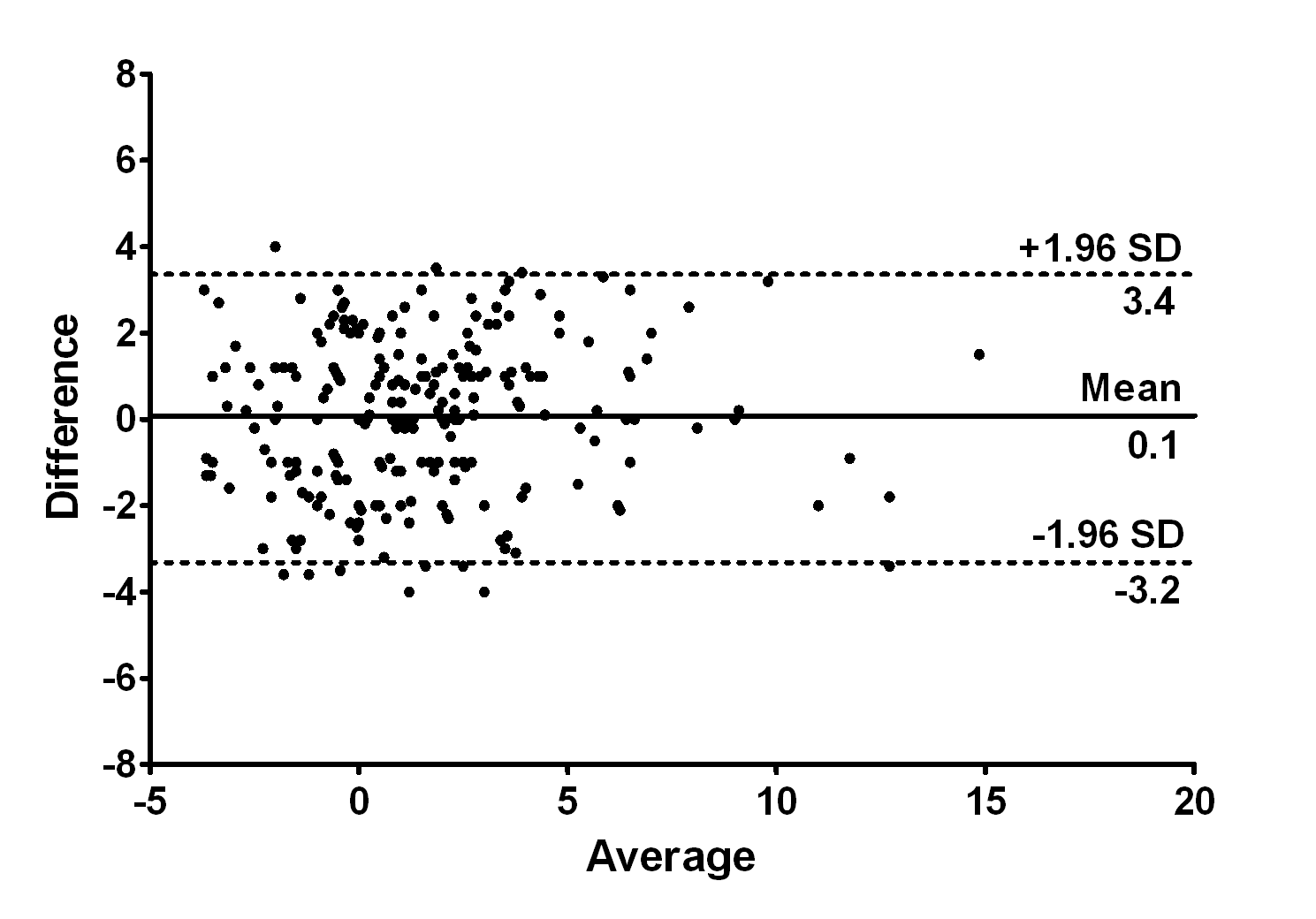

Supplement: S1 Fig — (TIF) [file pone.0122693.s001.tif]
