# Supplementary material for: Spinal Radiographic Progression in Patients with Ankylosing Spondylitis Treated with TNF-α Blocking Therapy: A Prospective Longitudinal Observational Cohort Study
Source: PLoS One. 2015 Apr 16;10(4):e0122693. doi: 10.1371/journal.pone.0122693 (PMC4400173; doi:10.1371/journal.pone.0122693)
Supplement: S1 Table — (PDF) [file pone.0122693.s002.pdf]

S1 Table. Baseline characteristics of AS patients with available 2, 4, or 6 years mSASSS data.

	With 2 years data (n=151)	With 4 years data (n=98)	With 6 years data (n=50)
**Male gender**	105 (70)	72 (74)	37 (74)
**Age (yrs)**	42.0 ± 11.5	41.8 ± 11.0	41.9 ± 9.8
**Symptom duration (yrs)**	13 (1-53)	15 (1-47)	18 (2-36)
**Time since diagnosis (yrs)**	5 (0-44)	5 (0-28)	9 (0-26)
**HLA-B27+**	114 (76)	81 (83)	37 (74)
**BMI (kg/m^2)**	26.3 ± 4.0	26.2 ± 3.9	25.2 ± 4.3
**Smoking (yrs)**	12 (0-54)	11 (0-54)	15 (0-54)
**NSAID use**	112 (74)	78 (80)	47 (94)
**ASAS-NAID index (0-100)**	50 (0-100)	50 (25-100)	52 (37-100)
**BASDAI (0-10)**	6.1 ± 1.6	5.8 ± 1.7	6.1 ± 1.5
BASDAI ≥6	71 (47)	40 (41)	21 (42)
**ASDAS$_{CRP}$**	3.7 ± 0.8	3.8 ± 0.8	3.9 ± 0.7
ASDAS >3.5	87 (58)	61 (64)	34 (69)
**Physician's GDA (0-10)**	4 (0-8)	5 (0-8)	5 (1-8)
Physician's GDA >6	28 (19)	25 (26)	14 (29)
**Patient's GDA (0-10)**	7 (1-10)	7 (1-10)	7 (1-10)
Patient's GDA >6	93 (62)	52 (54)	29 (58)
**CRP (mg/L)**	12 (1-82)	15 (1-99)	14 (1-82)
CRP >10 mg/L	81 (54)	61 (63)	33 (67)
**ESR (mm/hr)**	19 (1-101)	21 (2-101)	22 (2-101)
ESR >20 mm/hr	70 (47)	53 (55)	28 (57)
**mSASSS (range 0-72)**	10 (0-70)	13 (0-68)	11 (0-70)
**Presence of syndesmophytes**	84 (56)	59 (60)	27 (54)

Values are presented as number of patients (%), mean ± SD, or median (range).
AS: ankylosing spondylitis; HLA: human leukocyte antigen; BMI: body mass index; NSAID: non-steroidal anti-inflammatory drug; ASAS: Assessment of SpondyloArthritis international Society; BASDAI: Bath AS Disease Activity Index; ASDAS: AS Disease Activity Score; GDA: global disease activity; CRP: C-reactive protein; ESR: erythrocyte sedimentation rate; mSASSS: modified Stoke AS Spine Score.
